# Aortic valve replacement in patients with an anomalous left circumflex artery

**DOI:** 10.1093/icvts/ivab207

**Published:** 2021-07-29

**Authors:** Liam Bibo, Nicholas Bayfield, Damian Gimpel, Jurgen Passage

**Affiliations:** 1 Department of Cardiothoracic Surgery, Sir Charles Gairdner Hospital, Nedlands, WA, Australia; 2 School of Medicine, University of Notre Dame, Fremantle, WA, Australia

**Keywords:** Aortic valve replacement, Anomalous left circumflex, Cardiothoracic surgery

##  

## Reviewer information

Interactive CardioVascular and Thoracic Surgery thanks Sertac M. Cicek and the other, anonymous reviewer(s) for their contribution to the peer review process of this article.

